# Trend in overall survival from the start of first-line chemotherapy in patients with metastatic urothelial carcinoma

**DOI:** 10.1093/jjco/hyad151

**Published:** 2023-10-26

**Authors:** Shoma Yamamoto, Minoru Kato, Taisuke Matsue, Nao Yukimatsu, Yuji Takeyama, Taiyo Otoshi, Takeshi Yamasaki, Katsuyuki Kuratsukuri, Junji Uchida

**Affiliations:** Department of Urology, Graduate School of Medicine, Osaka Metropolitan University, Osaka, Japan; Department of Urology, Graduate School of Medicine, Osaka Metropolitan University, Osaka, Japan; Department of Urology, Graduate School of Medicine, Osaka Metropolitan University, Osaka, Japan; Department of Urology, Graduate School of Medicine, Osaka Metropolitan University, Osaka, Japan; Department of Urology, Ishikiri Seiki Hospital, Osaka, Japan; Department of Urology, Graduate School of Medicine, Osaka Metropolitan University, Osaka, Japan; Department of Urology, Graduate School of Medicine, Osaka Metropolitan University, Osaka, Japan; Department of Urology, Graduate School of Medicine, Osaka Metropolitan University, Osaka, Japan; Department of Urology, Graduate School of Medicine, Osaka Metropolitan University, Osaka, Japan

**Keywords:** avelumab, chemotherapy, enfortumab vedotin, metastatic urothelial carcinoma, pembrolizumab

## Abstract

New approaches involving immune checkpoint inhibitors and antibody-drug conjugates prolong overall survival in patients with metastatic urothelial carcinoma. However, the access to such systemic therapy in clinical practice is suboptimal, and whether these agents improve overall survival in patients with metastatic urothelial carcinoma over time remains unclear. Hence, we investigated the overall survival trend from the initiation of first-line therapy with these agents to identify changes due to the medication and time of treatment initiation. We retrospectively evaluated 195 patients from a single center. They were treated with chemotherapy, pembrolizumab, or avelumab or enfortumab vedotin. The treatment was categorized into chemotherapy, pembrolizumab or avelumab/enfortumab vedotin period. The new agents prolonged overall survival from the start of first-line therapy. Furthermore, sequential treatment with these agents in real-world clinical practice has been reported to prolong overall survival. These study results will have major implications when a new first-line therapy is approved in the future.

## Teaser

The prevalence of sequential treatment in the real-world practice of metastatic urothelial carcinoma was investigated. Overall survival was found to have increased over time from the initiation of first-line therapy.

## Introduction

Cisplatin-based chemotherapy has been the gold standard as the first-line treatment of metastatic urothelial carcinoma (mUC), and a 2000 clinical trial comparing gemcitabine plus cisplatin and methotrexate, vinblastine, doxorubicin, and cisplatin reported the median overall survival (OS) was 14 months (1). Pembrolizumab was approved in November 2017 as second-line therapy for patients showing disease progression after platinum-based chemotherapy, and the 2017 KEYNOTE-045 trial reported that the OS from the initiation of pembrolizumab was 10.3 months (2). Later, maintenance therapy with avelumab prolonged OS in patients who did not show disease progress after platinum-based chemotherapy, and the median OS from the start of maintenance therapy in avelumab-treated patients was 21.4 months (3). Recently, new agents such as enfortumab vedotin (EV), erdafitinib and sacituzumab govitecan have been approved by the US Food and Drug Administration for patients showing disease progression after treatment with immune checkpoint inhibitors (ICIs) (4–6). Ideally, the estimated median OS from the start of first-line therapy would be ~26.5 and 16–18 months, respectively, among avelumab- and pembrolizumab-treated patients (7). However, real-world studies have reported a lack of optimal receipt of systemic therapies (8). This low rate of receipt of subsequent therapy is attributable to the characteristic of rapid disease progression in patients with mUC as well as the healthcare system. In fact, few studies have investigated the effects of new therapeutic agents on OS after the initiation of first-line chemotherapy in real-world settings. In other words, there is a gap between the findings of clinical trials and real-world clinical practice. Hence, this study was aimed at investigating trends in OS and sequential treatment allocation in patients who received first-line platinum-based chemotherapy for mUC.

## Patients and methods

Patients with mUC who received first-line platinum-based chemotherapy at the Osaka Metropolitan University Hospital between April 2009 and August 2022 were enrolled. This retrospective, single-center study was conducted in accordance with the Declaration of Helsinki and was approved by the Ethics Committee of Osaka Metropolitan University Hospital (no. 2021-090, 4105). Overall, 195 patients were evaluated. OS was defined as the time from the start of the first-line chemotherapy and was calculated per the following two aspects: based on the anticancer agent used and on the time when the first-line treatment was initiated to observe the trend in OS so as to identify changes due to the medication and the time of treatment initiation. For the analysis based on the anticancer agent used, the patients were divided into the following three groups: chemotherapy, pembrolizumab and avelumab groups. The chemotherapy group was defined as the group of patients who received only chemotherapy during treatment. The pembrolizumab and avelumab groups were defined as the groups of patients who received any of these agents during treatment. The time of first-line chemotherapy initiation was classified into the following three periods based on regulatory approval in Japan: chemotherapy period (April 2009–June 2017; P1), pembrolizumab period (July 2017–December 2020; P2) and avelumab and EV period (January 2021–August 2022; P3). The last observation date was set for the end of July 2023. The patients’ data were statistically analyzed using GraphPad Prism version 9 (GraphPad Software, Inc., San Diego, CA, USA). OS was calculated using the Kaplan–Meier method, and the log-rank test was used to determine differences. Fisher's exact test or chi-square test was used to compare categorical variables. A *P* value of <0.05 was considered statistically significant.

## Results

### Patients’ backgrounds

The chemotherapy, pembrolizumab and avelumab included 82, 98 and 15 patients, respectively. In the analysis based on the time of initiation of the first-line therapy, the P1, P2 and P3 groups included 78, 79 and 38 patients, respectively. The distribution of clinical parameters was well-balanced in categories such as sex, age, anemia, tumor origin, metastatic sites and radical surgery, except for Eastern Cooperative Oncology Group Performance Status (ECOG PS) (Table 1). The avelumab group had better ECOG PS than the remaining groups.

**Table 1:** Patients' characteristics

Characteristics (*n* = 195)		Number of patients			
		Chemotherapy (*n* = 82)	Pembrolizumab (*n* = 98)	Avelumab (*n* = 15)	*P* values
Sex (%)	Male	64 (78)	67 (68)	10 (67)	0.3
	Female	18 (12)	31 (32)	5 (33)	
Age, years	Median (range)	73 (32–95)	73 (37–86)	70 (56–84)	0.51
EOCG PS (%)	0	66 (80)	70 (71)	15 (100)	0.03^*^
	≥1	16 (20)	28 (29)	0 (0)	
Hb (%)	≥10 g/dL	69 (84)	66 (67)	11 (73)	0.03^*^
	<10 g/dL	13 (16)	32 (33)	4 (27)	
Primary tumor sites (%)	Bladder	47 (57)	49 (50)	8 (53)	0.39
	Upper urinary tract	34 (41)	43 (44)	7 (47)	
	Both	1 (2)	6 (6)	0 (0)	
Metastatic site (%)	Lymph node	44 (54)	49 (50)	11 (73)	0.97
	Lung	34 (41)	33 (34)	6 (40)	
	Bone	21 (26)	21 (21)	3 (20)	
	Liver	17 (21)	17 (17)	2 (13)	
Surgical treatment (%)	Cystectomy	22 (27)	31 (32)	3 (20)	0.85
	Cystectomy+nephroureterectomy	0 (0)	2 (2)	0 (0)	
	Nephroureterectomy	22 (27)	25 (26)	5 (33)	
	Transurethral resection	14 (17)	17 (17)	2 (13)	
	None	24 (29)	23 (23)	5 (33)	

ECOG-PS, Eastern Cooperative Oncology Group performance status; Hb, hemoglobin.

*P* values are calculated with three groups comparison. ^^*^^P < 0.05

### Patients’ survival

**Figure 1:**
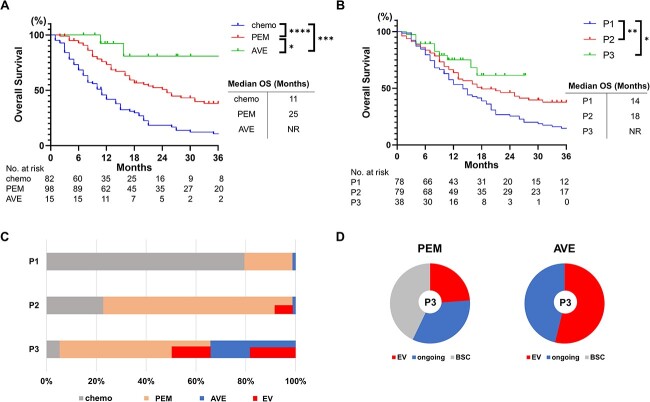
Trends in OS by anticancer agent (A) and period (B). EV is used for some patients in the PEM and AVE group. (C) The proportion of therapeutic agents in each period. (D) Treatment breakdown in the patients who received PEM or AVE in P3 at the time of data cutoff. Patients were either treated with EV, best supportive care, or continued ICI treatment (ongoing). OS, overall survival; ICI, immune checkpoint inhibitor; chemo, chemotherapy; PEM, pembrolizumab; AVE, avelumab; EV, enfortumab vedotin; BSC, best supportive care; NR, not reached; chemotherapy period (P1: April 2009–June 2017), PEM period (P2: July 2017–December 2020), AVE and EV period (P3: January 2021–August 2022), ^*^P < 0.05; ^*^^*^P < 0.01; ^*^^*^^*^P < 0.001; ^*^^*^^*^^*^P < 0.0001.

A significant difference in OS was observed between pembrolizumab- and chemotherapy-treated patients [hazard ratio (HR) = 0.44, 95% confidence interval (CI): 0.31–0.63, *P* < 0.0001], avelumab- and chemotherapy-treated patients (HR = 0.11, 95% CI: 0.062–0.19, *P* = 0.0001) and avelumab- and pembrolizumab-treated patients (HR = 0.26, 95% CI: 0.12–0.56, *P* = 0.037) (Fig. 1A).

Analysis by periods showed that OS was significantly prolonged in P2 compared with that in P1 (HR = 0.62, 95% CI: 0.43–0.89, *P* = 0.009) and in P3 compared with that in P1 (HR = 0.47, 95% CI: 0.28–0.78, *P* = 0.015). However, no difference was observed in OS between P2 and P3 (HR = 0.66, 95% CI: 0.36–1.20, *P* = 0.21) (Fig. 1B). In P1, the majority of patients (80%) belonged to the chemotherapy group (including those who received second-line taxane-based chemotherapy but not ICIs or EV), and the majority of patients received ICIs (pembrolizumab and avelumab) in P2 and P3 (77 and 95%, respectively). Overall, the proportion of patients who received ICIs increased over time, as shown in Fig. 1C (*P* = 0.005). In P3, when third-line treatment with EV was available, EV was administered to 24% of the pembrolizumab-treated patients and 54% of the avelumab-treated patients (Fig. 1C). Importantly, all the patients in avelumab group with disease progression received EV, while 43% of patients in the pembrolizumab group received best supportive care in P3 (Fig. 1D). We believe this gap in sequential treatment is due to the difference in tumor characteristics between those who received pembrolizumab and avelumab.

## Discussion

While a pivotal study has shown that new agents prolonged OS compared with the standard of care in patients with mUC who were eligible for clinical trials, it is important to determine whether this advantage could be obtained in real-world practice in the patients including those ineligible for clinical trials due to a deteriorated PS, rapid disease progression and other reasons. Although the therapeutic benefit of pembrolizumab can be expected irrespective of the objective response and number of cycles of first-line chemotherapy (9), we have previously shown that excessive cycles of first-line chemotherapy may not lead to improved OS, especially in the era of switching maintenance therapies and EV (10). In the present study, the transition rate of ICI treatment increased over time, while the prevalence of the treatment with EV was only 24% in the pembrolizumab group in P3, and 43% of patients received best supportive care. In addition, no significant prolongation in OS was observed between P2 and P3. This unexpected result might be because some patients who received ICIs (especially pembrolizumab) could not receive EV owing to rapid disease progression or other reasons. With the current access to EV as a sequential treatment, it is clinically important not to miss the opportunity for sequential treatment, except for patients who cannot continue the treatment owing to their declining general condition. Many clinical trials to investigate the benefit of combination therapy using ICIs vs platinum-based chemotherapy are ongoing. If the primary endpoints of these trials are achieved, the results of this study, especially the OS in P3, will have important implications as results achieved in a historical cohort in real-world clinical practice when new first-line therapies are approved in the future.

This study was limited by its retrospective nature, the small number of cases in a single institution and immortal time bias. Study limitations also include the lack of a multivariate analysis to minimize variation among the patients owing to the sample size. Prognostic factors such as anemia and poor PS may influence the feasibility of sequential therapy. However, this study showed an improvement in OS in patients with mUC treated with new treatment agents and because of the current prevalence of sequential treatment in real-world practice. This might be because that the ICI contributed to better access to the second-line therapy. The results in the present study may indicate the importance of not missing the appropriate opportunity to receive sequential treatments for mUC.

## Abbreviations & Acronyms

mUC: metastatic urothelial carcinoma

OS: overall survival

EV: enfortumab vedotin

ICIs: immune checkpoint inhibitors

ECOG PS: Eastern Cooperative Oncology Group Performance Status

HR: hazard ratio

CI: confidence interval
